# Epidermal Notch signalling: differentiation, cancer and adhesion

**DOI:** 10.1016/j.ceb.2008.01.010

**Published:** 2008-04

**Authors:** Fiona M Watt, Soline Estrach, Carrie A Ambler

**Affiliations:** 1 Cancer Research UK Cambridge Research Institute, Li Ka Shing Centre, Robinson Way, Cambridge CB2 0RE, UK; 2 Wellcome Trust Centre for Stem Cell Research, Tennis Court Road, Cambridge CB2 1QR, UK

## Abstract

The Notch pathway plays an important role in regulating epidermal differentiation. Notch ligands, receptors and effectors are expressed in a complex and dynamic pattern in embryonic and adult skin. Genetic ablation or activation of the pathway reveals that Notch signalling promotes differentiation of the hair follicle, sebaceous gland and interfollicular epidermal lineages and that Notch acts as an epidermal tumour suppressor. Notch signalling interacts with a range of other pathways to fulfil these functions and acts via RBP-Jκ dependent and independent mechanisms. The effects on differentiation can be cell autonomous and non-autonomous, and Notch contributes to stem cell clustering via modulation of cell adhesion.

## Introduction

Mammalian epidermis, the outermost layer of the skin, is maintained by stem cells that self-renew and produce progeny that undergo terminal differentiation to generate the interfollicular epidermis (IFE), hair follicles (HF) and sebaceous glands (SG) [1–3]. There appear to be distinct populations of stem cells within the IFE, SG and HF, and the differentiation pathway selected by their progeny is largely determined by local microenvironmental signals [2,3].

In recent years there has been considerable progress in identifying the signalling pathways that regulate the epidermal stem cell compartment [1,3]. Many of these pathways are evolutionarily conserved and were originally defined by their role in Drosophila embryonic development. In this article we discuss the diverse ways in which Notch signalling regulates epidermal differentiation.

**Figure 1:**
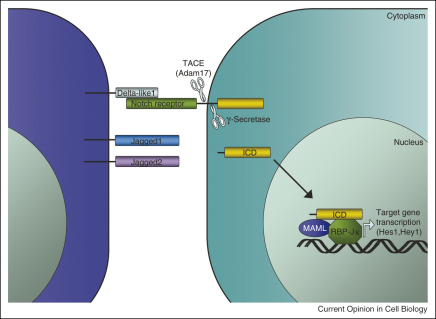
The core Notch signalling pathway. ICD: intracellular domain; MAML: Mastermind-like. Based on reference [4].

The core Notch signalling pathway is shown in Figure 1 [4–6]. Signalling occurs over a short range because the receptors and ligands are transmembrane proteins. In vertebrates there are four Notch receptors. The ligands are subdivided into two classes, Delta or Delta-like (Dll) and Serrate or Jagged, which differ because of the presence in Jagged ligands of a cysteine-rich domain that is lacking in the Dll ligands.

When ligand binds the Notch receptor, Notch undergoes two proteolytic cleavage events (Figure 1) [4–6]. The first is mediated by an ADAM family metalloproteinase (ADAM10 or ADAM17, also known as TACE, for TNF-α-converting enzyme). This generates a substrate for the second proteolytic event mediated by a γ-secretase complex. As a result of the second event the Notch intracellular domain (NICD) is released from the plasma membrane; it enters the nucleus and forms a complex with the DNA binding protein RBP-Jκ (also known as CSL, CBF1, Su(H) and LAG-1). The co-activator Mastermind (Mastermind-like (MAML) 1–3 in vertebrates) is recruited to the complex and activates transcription of target genes, including Hes and Hey family genes.

While the core signalling pathway is simple, there is considerable complexity in the way that signalling is regulated through post-translational modifications of receptors and ligands [4–6]. The Fringe glycosyl transferase, resident in the Golgi, adds carbohydrate chains to the extracellular domain of Notch, thereby altering the ability of ligands to activate the receptor. Furthermore, the ligands, like the receptors, undergo controlled proteolysis and there is some indication that the cleaved ligand intracellular domains also participate in signalling.

## Sites of Notch pathway activity

**Figure 2:**
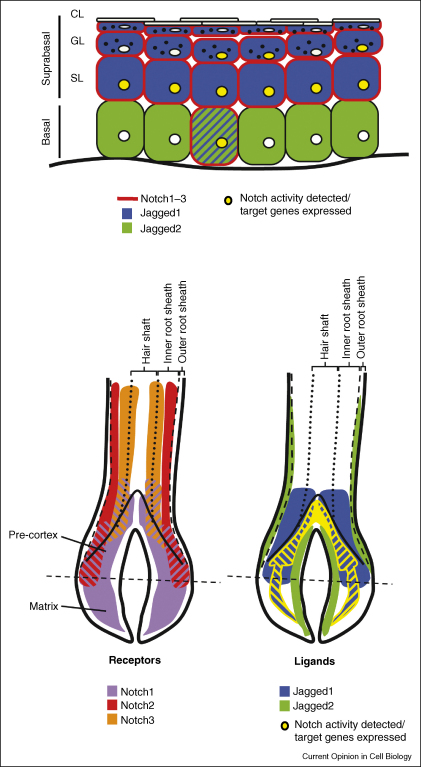
Expression of Notch ligands and receptors. Neonatal interfollicular epidermis (top panel) and hair follicles (bottom panel) are shown. Hatched shading indicates co-expression. CL: cornified layer; GL: granular layer; SL: spinous layer. Adapted from references [7^••^,11].

There is well-documented expression of Notch1, Notch2 and Notch3 [7^••^,8–13] in the IFE, and the expression of Notch4 has also been reported [10]. Most studies find that Notch expression is upregulated in the suprabasal cells of the IFE (Figure 2). Notch receptors are also expressed within the hair follicle. They are found both in the base of the follicle (matrix and precortex), which is the site of active proliferation and commitment to terminal differentiation, and more distally [8,9,11,12]. Thus, in both the IFE and hair follicle the sites of Notch expression coincide with cells that are initiating or are undergoing terminal differentiation.

The Notch ligands detected in the epidermis are Delta-like 1 (Dll1; Delta1), Jagged1 and Jagged2. In the interfollicular epidermis Jagged1 is primarily expressed in the suprabasal layers but has also been detected in some basal cells; Jagged2 is expressed in the basal layer (Figure 2) [10–13,14^••^]. In the hair follicle Jagged2 is expressed in the follicle bulb cells next to the dermal papilla and in the basal layer of the outer root sheath [8,12]. Jagged1 is expressed in the suprabasal layers of the upper outer root sheath and bulb pre-cortex [12,14^••^]. Thus, there are complementary patterns of expression of Jagged1 and Jagged2, both in IFE and at the base of the HF (Figure 2).

By *in situ* hybridisation Dll1 has been detected in the basal layer of foetal and adult human IFE [13,15], and antibody staining suggests that Dll1 expression is highest in stem cell clusters [15]. In mouse skin Dll1 is reported to be expressed in embryonic but not postnatal epidermis, with expression confined to the mesenchymal cells that will form the dermal papilla [8,12]. However, recent analysis of E18.5 mouse embryonic skin in which the β-galactosidase gene is knocked into the Dll1 locus has provided evidence that Dll1 is not only expressed in the dermal papilla but also in the epithelial cells at the base of the hair follicle and in clusters of cells in the basal layer of the IFE [16^••^]. Since deletion of Dll1 results in a phenotype in adult mouse IFE it is possible that the ligand continues to be expressed postnatally [16^••^].

In addition to documenting expression of Notch pathway components, several studies have examined pathway activation (Figure 2). This is important because even in cells expressing ligand and receptor the pathway may be inactivated, for example through expression of the vertebrate homologues of Drosophila Fringe, Lunatic, Radical and Manic [8,13]. The HF pre-cortex and inner root sheath progenitors are labelled with antibodies to NICD, indicative of Notch1 activation [7^••^,11,14^••^,17^••^,18]. NICD is also detected in some basal cells of the IFE and throughout the suprabasal (spinous and granular) layers [7^••^,11,14^••^,19,20]. While NICD labelling identifies cells that have received a Notch signal, there is specificity in the transcriptional response, as revealed by the patterns of expression of different Hes and Hey genes in embryonic and postnatal epidermis [7^••^,21^•^,22^•^].

Notch activation can also be monitored using transgenic reporter lines. When a GFP transgene is expressed under the control of an RBP-J response element [14^••^] or the endogenous Hes1 promoter [22^•^], GFP is detected in the pre-cortex of growing (anagen) hair follicles. GFP is also detected in the cuticle of the inner root sheath, the outer root sheath and the dermal papilla, in suprabasal and a few basal cells of the IFE, in good agreement with the sites where NICD is detected [14^••^]. In a different approach, Cre recombinase expression is controlled by ligand-induced proteolysis of the Notch1 transmembrane tether, and Cre-mediated β-galactosidase expression is used to identify the descendents of cells that have experienced Notch1 activation [23^•^]. In developing IFE β-galactosidase is detected exclusively in the suprabasal layers and not in the basal layer, and β-galactosidase is almost undetectable in postnatal IFE, arguing against Notch1 activity in the IFE stem cell compartment [23^•^].

The conclusion from these studies is that there is dynamic expression of Notch pathway components in embryonic and adult epidermis and that the pathway is primarily active in cells of the IFE and HF that are committed to, or undergoing, terminal differentiation.

## Consequences of deleting or activating pathway components

Given the number of Notch receptors and ligands expressed in the epidermis, complete inactivation of signalling is only achieved by targeting common Notch pathway components, such as γ-secretase, RBP-Jκ or Mastermind-like (Figure 1). Deletion of γ-secretase in embryonic ectoderm and postnatal anagen (growing) follicles has shown that Notch activity is not required to initiate HF morphogenesis, but in postnatal skin lacking the enzyme mature sebocytes are absent, the IFE is hyperproliferative and HF convert into cysts of cells undergoing IFE differentiation [11]. Conditional ablation of the RBP-Jκ gene [7^••^,24] or expression of a dominant negative form of Mastermind-like 1 [25^••^] causes a similar phenotype to γ-secretase deficiency, with defective HF maturation, impaired SG differentiation, epidermal hyperkeratinisation and epidermal cyst formation.

Deletion of Notch2, Notch3 or Notch4 alone does not have any reported effects on the epidermis [11,26,27]. On deletion of Notch1 the HF form normally in the embryo, but their morphology is disturbed and adult epidermis lacks mature sebocytes [11,18]. Combined deficiency of Notch1 and Notch2 mimics loss of γ-secretase in the HF, while combined loss of Notch1, Notch2 and Notch3 resembles the complete γ-secretase deficiency phenotype [11].

Epidermal deletion of Jagged1 leads to conversion of HF into cysts of IFE, with thickening of the IFE, thereby resembling the consequences of deleting Notch1 [14^••^]. Deletion of Dll1 results in a delay in the first postnatal wave of hair growth, but no further effects on the HF [16^••^]. However, there is increased proliferation in Dll1-deficient IFE and disturbed expression of differentiation markers [16^••^]. Jagged2-deficient skin has no overt abnormalities of the hair follicles and interfollicular epidermis (Lee J and Kopan R, personal communication). These results suggest that whereas Jagged1 is the primary Notch ligand involved in regulating hair follicle differentiation, Dll1 contributes to the control of IFE proliferation and differentiation.

Complementing the genetic ablation approach are studies of the effects of activating the pathway by overexpressing NICD in different epidermal layers. Overexpression of NICD in the suprabasal layers of the IFE and the HF inner root sheet leads to expansion of the differentiated cell compartment of the IFE, disturbed differentiation of HF lineages and hair loss [28]. Overexpression of NICD in the basal layer of the epidermis, SG and HF results in expansion of the IFE spinous layers and reduced granular cell differentiation [7^••^,14^••^]. There is also expansion of the base of the hair follicle, sebaceous gland enlargement and abnormal clumping of HF [14^••^]. Newborn mice in which NICD is overexpressed in the basal layer have severe skin blistering associated with reduced expression of the α6β4 integrin [7^••^].

The conclusion from these studies is that while Notch signalling is not required for embryonic development of the epidermis, it is essential for postnatal maintenance of the hair follicles and SG and regulates terminal differentiation within the IFE. Activation of Notch is not sufficient to induce ectopic hair follicles but does promote differentiation of the HF lineages.

## Non-cell autonomous effects

**Figure 3:**
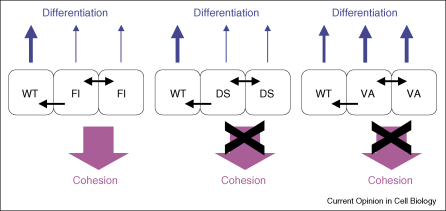
Model showing cell autonomous and non-cell autonomous roles of Dll1 in human IFE reconstituted in culture. Reproduced from reference [40^••^] with permission of the Company of Biologists. Keratinocytes are shown as wild type (WT) or overexpressing full-length Dll1 (Fl), Dll1 lacking the cytoplasmic domain (DS) or Dll1 with a point mutation in the C-terminal PDZ domain (VA). The symbol (←) represents signal from Delta-expressing cell to wild-type cell; the symbol (↔) represents reciprocal signalling between two Delta-expressing cells. The strength of the differentiation signal is represented by the thickness of the vertical arrows. Intercellular adhesion (cohesion) is shown as being promoted (arrow) or not (cross through arrow).

One of the most important aspects of Notch signalling during Drosophila development is that it involves non-cell autonomous effects [4]. The prime examples are lateral inhibition, in which Notch signalling amplifies small differences within a population of cells, and boundary formation, in which Notch signalling between two populations of cells can cause those cells to segregate. There is clear evidence that Notch has non-cell autonomous effects on epidermal differentiation. In human IFE reconstituted in culture, cells that overexpress Dll1 form clusters; cells within the cluster do not undergo terminal differentiation themselves but stimulate neighbouring cells with lower levels of Dll1 to initiate terminal differentiation (Figure 3; [15]). Overexpression of NICD in a single HF lineage triggers abnormal differentiation of the neighbouring cell types [29], and ectopic activation of Notch1 in postmitotic cells within the nail stimulates proliferation of transgene-negative cells within the tissue [19].

Examples of non-cell autonomous signalling extend to interactions between keratinocytes and other cell types. Wounding of Notch1-deficient cornea causes transdifferentiation into epidermis, a process that involves secretion of fibroblast growth factor-2 (FGF-2) by the epithelium, resulting in vascularisation and remodelling of the underlying stroma [30^••^]. Notch signalling within melanocytes is required to maintain the melanocyte stem cell compartment [22^•^,31^•^]. Intriguingly, Notch1 deletion in hair follicle epithelium also leads to a decrease in melanocyte number, and this is attributable to decreased epithelial expression of Kit ligand, an essential survival and proliferation factor for melanocytes [17^••^].

## Notch signalling and cancer

Classically, cells with high levels of Notch activity suppress the differentiation of their neighbours [4], and in some mammalian tissues activation of Notch is associated with tumours, the best characterised example being gain-of-function Notch1 mutations in human T cell acute lymphoblastic leukaemia/lymphoma [32]. By contrast, consistent with the finding that Notch signalling promotes epidermal terminal differentiation, Notch1 acts as a tumour suppressor in the skin [33]. Epidermis in which Notch signalling is inhibited by deletion of Notch1 or overexpression of a dominant negative inhibitor of Mastermind-like 1 develops spontaneous squamous cell carcinomas [25^••^,33]. Notch1 deletion also increases the sensitivity of the epidermis to developing tumours in response to activated Ras [33,34^••^]. Spontaneous tumour formation has not been observed in Jagged1-deficient epidermis [14^••^]. However, tumours do develop in aged mice that lack Dll1 in the epidermis [16^••^].

Notch is reported to be a p53 target gene [34^••^]. Conversely, loss of Numb, which is a Notch antagonist, leads to attenuation of the p53 tumour suppressor pathway [35]. However, ablation of p53 does not influence the Notch deficiency phenotype in the hair follicle [17].

## Growth arrest and initiation of terminal differentiation

The mechanism by which Notch activation induces IFE terminal differentiation has been examined primarily in cultured epidermal cells (keratinocytes). While it clearly involves canonical signalling via RBP-Jκ [7^••^,15,36], there is also a complex interaction with other pathways. Keratinocytes that undergo terminal differentiation withdraw from the cell cycle; however, growth arrest and terminal differentiation are under separate control. Growth arrest involves Notch1-mediated induction of p21 [36]. While RBP-Jκ binds to the p21 promoter [36], activation of p21 is also dependent on positive regulation of NFAT activity by activated Notch1, and an interaction between Notch1 and calcineurin-NFAT signalling is involved in HF maintenance [37].

Notch-induced differentiation involves Notch-dependent suppression of p63 expression through a mechanism that is independent of cell cycle withdrawal [38]. p63 negatively regulates Hes1 gene expression and counteracts the differentiation-promoting activity of Notch activation, suggesting that a mututal antagonism between Notch and p63 is involved in epidermal homeostasis [38].

In neonatal RBP-J null epidermis there is repression of spinous and granular layer markers [7^••^]. RBP-J is essential for mediating both induction of spinous layer genes and repression of basal genes. It has been reported that Hes1, which is expressed in the spinous layers, mediates spinous gene induction but not repression of basal layer genes, raising the possibility of involvement of other Hes/Hey family genes [21^•^]. However, this contrasts with an earlier report [38]. There is evidence that caspase 3 is a transcriptional Notch1 target that plays a role commitment of embryonic keratinocytes to terminal differentiation [20], although this has not been confirmed *in vivo* [7^••^].

As already described, in cultured primary human keratinocytes overexpression of Dll1 does not promote differentiation within the expressing cells (Fl Dll1 in Figure 3) but triggers terminal differentiation of neighbouring cells (non-cell autonomous; [15]); indeed Dll1 expression is used as a marker of cultured human epidermal stem cells [39^•^]. By contrast, overexpression of a Dll1 mutant in which the C-terminal PDZ domain is inactivated (VA Dll1 in Figure 3) stimulates terminal differentiation in the expressing cells, indicative of a cell autonomous effect [40^••^]. The ability of the PDZ mutant to activate a Hes1 luciferase reporter is substantially higher than that of wild-type Dll1 [40^••^]. This suggests that autonomous Notch-induced terminal differentiation may require a stronger signal than non-cell autonomous induction of differentiation (Figure 3).

In addition to the possibility that signal strength affects differentiation, there is indirect evidence that different Notch ligands exert different effects on differentiation [16^••^]. Thus, in culture Dll1 null but not Jagged1 null keratinocytes exhibit decreased integrin expression and this correlates with a higher tendency of Dll1 null cells to initiate terminal differentiation [16^••^].

It is clear from these studies that the mechanisms by which Notch signalling regulate terminal differentiation are incompletely understood. However, the role of Notch in growth arrest is different from its role in initiation of terminal differentiation; there are non-cell autonomous effects that may be linked to signal strength; and it is possible that different Notch ligands elicit different cellular responses.

## Lineage selection

In addition to contributing to the decision of a cell to self-renew or initiate terminal differentiation, Notch signalling affects the choice of which differentiation programme is selected. In doing so, Notch interacts with at least two other signalling pathways, involving Vitamin A (retinoic acid) and Wnt. Vitamin A levels have long been known to influence the programme of terminal differentiation that occurs in the suprabasal layers of multilayered epithelia: high levels are associated with ‘nonkeratinised’ (lacking cornified layers; see Figure 2) epithelia, such as the cornea, and lower levels with ‘keratinised’ epithelia, such as the epidermis. Cellular retinol binding protein 1 (CRBP1), which is required for retinol metabolism into retinoic acid, has recently been identified as a Notch target gene [30^••^]. The conversion of corneal to epidermal epithelium observed in Notch1-deficient mice is also observed on deletion of CRBP1 [30^••^].

**Figure 4:**
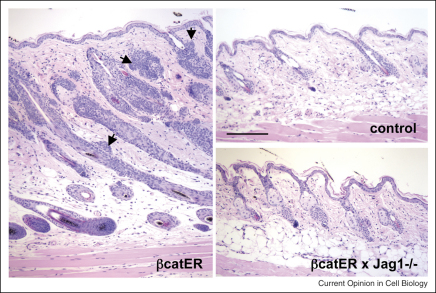
Requirement of Jagged1 for induction of ectopic hair follicles by β-catenin. Reproduced from reference [14^••^] with permission of the Company of Biologists. Activation of β-catenin in a wild-type background (βcatER) induces ectopic hair follicle formation (arrows) and stimulates growth (anagen) of existing hair follicles. Anagen and ectopic hair follicle formation are blocked by epidermal deletion of Jagged1 (β-catER × Jag1^−/−^). The control is back skin from a Jag1 flox/flox mouse. Scale bar: 100 μm.

Activation of the Wnt pathway is required to form HF during embryogenesis and to maintain them postnatally. Activation of the pathway in adult epidermis results in the formation of ectopic follicles (Figure 4), whereas inhibition of the pathway leads to conversion of HF into cysts of IFE [1,3]. Several Notch pathway genes are upregulated by β-catenin activation in the epidermis [21^•^] and Jagged1 is a direct target gene of canonical Wnt signalling [14^••^]. Deletion of Jagged1 blocks β-catenin-induced ectopic HF formation (Figure 4) without preventing the ability of β-catenin to stimulate epidermal proliferation [14^••^]. Whereas overexpression of NICD does not induce ectopic HF formation it does enhance differentiation within β-catenin-induced follicles [14^••^].

While there is clear evidence for a positive effect of Wnt signalling on the Notch pathway there is also evidence for antagonism. In support of this, activation of Wnt signalling is observed in the tumours that form on epidermal overexpression of Mastermind-like 1 [25^••^]. One potential mechanism is via Notch1-mediated suppression of Wnt4 expression [41]. Another is binding of the Notch cytoplasmic domain to β-catenin, which negatively regulates β-catenin transcriptional activity [42]. However, adding further complexity, activation of Notch1 in differentiating nail cells results in ectopic activation of Wnt signaling [19], which places Notch upstream of Wnt.

We conclude that Notch, Wnt and Vitamin A are all part of the web of intersecting pathways that regulate epidermal lineage selection [3].

## Notch and adhesion

One final aspect of Notch signalling in the epidermis to consider is the regulation of cell adhesion (Figure 3). This is an important function of the pathway because the location of a cell determines the local microenvironmental signals it receives [3,15]. In epidermis reconstituted in culture by mixing cells with different levels of Dll1 expression, high Dll1 expression promotes keratinocyte cohesiveness, defined as the tendency of groups of cells to remain in contact with one another; this effect requires the cytoplasmic domain of Delta1 (see DS in Figure 3) and is independent of RBP-Jκ [15,43]. Cell mixing experiments do not distinguish autonomous from non-cell autonomous effects. However, Dll1 overexpression does exert a cell autonomous effect because it stimulates the spreading of individual keratinocytes on extracellular matrix [43].

Secreted forms of the extracellular domain of Jagged1 decrease extracellular matrix adhesion and migration of NIH3T3 cells; focal adhesion formation is reduced and formation of cadherin-mediated intercellular junctions is increased [44,45]. *In vivo*, Notch activation decreases expression of the α6β4 integrin [7^••^,36]. However, there is also evidence that NICD can activate integrin–ligand binding activity without affecting integrin levels [46]. Different Notch ligands can have different effects, since integrin expression is decreased in Dll1-null keratinocytes but increased in Jagged1-null cells [16^••^]. Reduced extracellular matrix adhesion is a potent terminal differentiation stimulus for cultured keratinocytes [47], and this raises the possibility that Notch ligand-specific effects on adhesion could explain why Dll1 differs from Jagged1 in its effects on differentiation [16^••^].

One mechanism by which Notch modulates keratinocyte adhesion is by suppressing expression of ROCK2 and MRCKα, effectors of RhoA and Cdc42, respectively [34^••^]. Combined knockdown of ROCK2 and MRCKα reduces integrin expression and cell motility, while upregulation suppresses differentiation and expands the stem cell compartment.

Another protein recently identified as playing a role in Notch-dependent stimulation of differentiation and cell adhesion (Figure 3) is syntenin [40^••^]. Syntenin binds to the Delta1 PDZ domain. Knockdown of syntenin in cells overexpressing Dll1 has the same effects on Notch signalling, epidermal differentiation and cell–cell adhesion as overexpressing Delta1 with a mutated PDZ binding domain (VA in Figure 3). Syntenin regulates endocytosis, and mutation of the Delta1 PDZ binding domain or knockdown of syntenin leads to rapid internalisation of Delta1, which is likely to contribute to stimulation of Notch signalling.

These studies point to a dual role of Notch-mediated changes in cell adhesion: determination of cellular location and regulation of terminal differentiation. The effect on differentiation can either be direct [47] or via exposing a cell to particular microenvironmental signals.

## Conclusions

Notch pathway functions in development can be subdivided into three categories [4]: lateral inhibition and boundary formation, as discussed earlier, and lineage decisions, in which cell fate is dependent on asymmetrical inheritance of Notch regulators. There is already evidence that Notch signals non-cell autonomously within the epidermis to regulate differentiation and can contribute to boundary formation by altering the adhesive properties of keratinocytes. However, in addition, expression of the cytoplasmic Notch inhibitor Numb in the IFE is consistent with a role for Notch in asymmetric fate determination [48^••^]. Therefore, the key functions ascribed to the pathway in Drosophila embryos are all recognisable in adult mammalian epidermis. Challenges for the future are to understand the complex interplay between Notch and other pathways; to explore the possibility that different levels of Notch signal have different outcomes; and to investigate Notch ligand-specific effects.

## References and recommended reading

Papers of particular interest, published within the annual period of review, have been highlighted as:

• of special interest
•• of outstanding interest
